# Antioxidant Activity and UHPLC-MS/MS Characterization of Polyphenol and Nicotine Content in Nicotiana Glauca Leaf Extracts: A Comparative Study of Conventional and Deep Eutectic Solvent Extraction Methods

**DOI:** 10.3390/plants13162240

**Published:** 2024-08-13

**Authors:** Reem Issa, Faisal Al-Akayleh, Lilian Alnsour, Tabarak R. Al-Sammarraie, Khaled W. Omari, Shady H. Awwad

**Affiliations:** 1 Department of Pharmaceutical Sciences, Pharmacological and Diagnostic Research Center (PDRC), Faculty of Pharmacy, Al-Ahliyya Amman University, Amman 19328, Jordan; l.alnsour@ammanu.edu.jo (L.A.); 202137088@ammanu.edu.jo (T.R.A.-S.); 2 Faculty of Pharmacy and Medical Sciences, Department of Pharmaceutics, Petra University, Amman 11196, Jordan; 3 College of Engineering and Technology, American University of the Middle East, Egaila 54200, Kuwait; khaled.omari@aum.edu.kw; 4 Department of Pharmaceutical Chemistry and Pharmacognosy, Faculty of Pharmacy, Applied Science Private University, Amman 11937, Jordan

**Keywords:** *Nicotiana glauca*, chromatography, mass spectrometry, antioxidants, deep eutectic solvents, sustainable extraction

## Abstract

The leaves of *Nicotiana glauca* (*N. glauca*; Solanaceae) plant are a known, major human health concern. This study investigated the antioxidant activity and polyphenols composition of aerial parts of *N. glauca* collected from its wild habitat in Jordan, using Methanol-Conventional (MC) and deep eutectic solvents (DES) extraction methods in addition to nicotine content determination using UHPLC. Our results showed that the MC extract contains fewer total phenols and flavonoid content than the 90% DES extract, (0.1194 ± 0.009 and 0.311 ± 0.020 mg/mL equivalent to gallic acid) and (0.01084 ± 0.005 and 0.928 ± 0.09 mg/mL equivalent to rutin), respectively. Moreover, this study showed that the prepared MC extract contain 635.07 ppm nicotine, while the 90% DES extract contain 1194.91 ppm nicotine. Extracts prepared using the MC and the DES methods exhibited weak antioxidant activities; the highest was a 33% inhibition rate (equivalent to ascorbic acid), obtained by the 90% DES extract,. The performed UHPLC-MS/MS analysis in this study also revealed the presence of variations in the detected compounds between the two extraction methods. Furthermore, this study found that environmentally friendly DES extraction of *N. glauca* produced higher phenol and flavonoid content than the MC method; this highlights the superior efficiency and environmental benefits of sustainable chemistry methods for extracting valuable phytoconstituents.

## 1. Introduction

Tobacco, which is derived from the leaves of the Nicotiana plant, is known for its abuse and is recognized as a major global health concern. However, it has been used in traditional medicine by Native Americans to treat respiratory, parasitic, and mental problems [1]. Later in Europe, the tobacco plant was enlisted in various pharmacopeias, with therapeutic applications in treating catarrh, colds, and fevers. It was also used as a digestion aid, a purgative, and a narcotic. Later in the 20th century, some reports suggested that tobacco might lower the risk of Alzheimer’s disease, Parkinson’s, and Tourette’s syndrome [2].

Several compounds, like alkaloids, steroids, tannins, and flavonoids, were isolated from Nicotiana species. Many of these metabolites are bioactive with reported anti-inflammatory, antitumor, antibacterial, and antioxidant activities [3]. For instance, *Nicotiana glauca* Graham (Solanaceae) was shown to contain anabasine as the major alkaloid in the methanolic extract of their leaves [4], which is known to possess antiparasitic activity [5]. A study by Ameya et al. (2017) revealed that *N. tabacum* L. contains pyridine alkaloids with antibacterial activity against biofilm-forming pathogens [6]. These alkaloids were used to treat strep throat caused by *Streptococcus pyogenes* [7] and showed activity against *Staphylococcus aureus*, *Enterococcus faecalis*, *Escherichia coli*, and *Pseudomonas aeruginosa* [8].

Moreover, several reports have highlighted the antioxidant activity of tobacco plant extracts, suggesting potential applications for various purposes. According to a recent study the methanol extract of *N. glauca* contains high levels of phenolic compounds, such as Chlorogenic acid and rutin [9]. These compounds were found to contribute to anti-inflammatory, anti-aging, and anticancer effects [3]. Another study from Saudi Arabia has demonstrated the antimicrobial effects of *N. glauca* against *E. coli* and *S. aureus*. The study concluded that the extracts from the leaves and flowers had the highest amounts of phytochemicals [10].

Conventional methods for extracting natural alkaloids and flavonoids, such as Soxhlet, maceration, percolation, and organic solvent extraction, are well-studied but have significant drawbacks. These techniques are time-consuming, inefficient, and often require large quantities of toxic, flammable, and non-biodegradable solvents, making them non-specific and not cost-effective. To address these issues, innovative solvents like deep eutectic solvents (DES) and natural deep eutectic solvents (NaDES) have been recently utilized [11,12,13,14]. As a subclass of ionic liquids (ILs), NaDES are considered less toxic, lower-cost, greener, and more efficient alternatives to both conventional organic solvents and ILs [15]. NaDES are usually prepared from a hydrogen bond donor and a hydrogen bond acceptor, which, when mixed in certain ratios, form a liquid at room temperature. Overall, the versatility, low toxicity, and environmentally friendly nature of NaDES make them attractive for a broad spectrum of industrial and research applications as solubilizers [16], drug delivery vehicles [17,18,19,20], stability enhancers [21], extraction and purification [22,23]. Additionally, NaDES themselves have been reported to possess antimicrobial [24], antioxidant [25], antibiofilm agents [26], and wound healing activity [17] among other beneficial effects. Choline chloride (a hydrogen bond acceptor) and malonic acid (a hydrogen bond donor) are natural compounds and are among the most commonly used substances for preparing NaDES [27].

In the context of the application of NaDES, this method was utilized for the extraction of polyphenols from *Citrus aurantium* L. peel [28]. The results showed enhanced recovery efficiency of polyphenols in the obtained extracts. Regarding tobacco plants, Hong et al. (2022) proposed the DES method for the extraction of solanesol from waste tobacco leaves [29]. Recently, cembranoid-type diterpenes compounds, known for their anticancer and antimicrobial effects, were extracted from tobacco flower waste using DES. Findings revealed the importance of green technologies in waste management and the extraction of bioactive natural compounds [30].

Hassan et al. (2014) conducted a phytochemical analysis of the *N. glauca* growing in Egypt [31]. Findings showed that the content of flavonoids in *N. glauca* was influenced by its habitat’s different conditions, which also affected the antioxidant activities. Therefore, research should take into consideration the use of medicinal plants relative to their composition of active and/or toxic metabolites collected from different regional areas.

This study aims to investigate the antioxidant activity and polyphenols composition of aerial parts of *N. glauca* species collected from its wild habitat in Jordan, using Methanol-Conventional (MC) and deep eutectic solvents (DES) extraction methods. This may provide information relevant to phenols content and antioxidant effect of the prepared extracts, revealing novel proposed uses with economic values ensuring the sustainable use of this plant species.

## 2. Materials and Methods

### 2.1. Plant Material

Fresh leaves of *N. glauca* were collected from widely grown plants in North Jordan, during the spring of 2022. The plant material was authenticated by an expert botanist in the Royal Botanical Gardens, Jordan. The voucher sample was deposited in the laboratory of Al-Ahliyya Amman University (Amman, Jordan). *N. glauca* leaves were dried under shade before the reduction in size using a conventional grinder and kept in a dark dry place at room temperature until used.

### 2.2. Methanol Conventional Extraction (MC)

An extract of the study plants was prepared using 50 g of dry plant material in 500 mL of methanol using the soaking method for 72 h at room temperature. This process was repeated twice, then the suspension was filtered and concentrated until a fine powder was obtained using Benchtop Manifold Freeze Dryer from Millrock Technology^®^.

### 2.3. Deep Eutectic Solvents (DES) Extraction

The DES were prepared from malonic acid and choline chloride in a 1:1 *w*/*w* molar ratio by physically mixing the two components gently on a hotplate to around 50–80 °C until a clear, homogeneous liquid was formed. The prepared DES mixture was mixed with deionized water to prepare three different extraction mixtures namely 30%, 70%, and 90% *v*/*v*. The cold extraction method was utilized by adding 5 g of the dry powder plant material in 25 mL of the DES extraction media. The plant material was soaked in the solvent for 72 h and then filtered to complete the extraction process.

### 2.4. Determination of Total Phenolic

The total phenolic content was measured using the Folin-Ciocalteu method as described by Alnsour et al., 2022 [32]. The phenolic content was determined calorimetrically at 765 nm. The total phenolic content (mg/mL) was determined as gallic acid equivalent. A stock solution of the plant extract was prepared at a concentration of 5 mg/mL. Serial dilutions were made, and an aliquot of each sample concentration (80 μL) was added to Folin–Ciocalteu (400 μL) reagent in a test tube, mixed with 7.5% sodium carbonate solution (320 μL). The solution was incubated in a dark place at 45 °C water bath for 30 min. Total phenolic content was expressed as gallic acid equivalent (mg/g), using the standard curve (Equation (1)):

y = 0.0049x + 0.0426, R^2^ = 0.9991 (1)

y = absorbance at 765 nm and x = concentration of total phenolic content gallic acid equivalent mg/mL.

### 2.5. Determination of Total Flavonoids

The determination of total flavonoids was performed using a colorimetric method based on the formation of a complex flavonoid–aluminum, measured at a wavelength of 510 nm using a UV-spectrophotometer as described by Ubaydee et al. (2022) [33]. The results were expressed as (mg/mL) equivalents to quercetin. Briefly, a stock solution of the plant extract at a concentration of 5 mg/mL was prepared. Serial dilutions were made, 1 mL of each concentration was added into (0.5 mL) AlCl3, (0.5 mL) NaNO2, (2 mL) NaOH, and (4 mL) distilled water. The mixture was incubated at room temperature for 15 min.

Total Flavonoid content was expressed as rutin equivalent (mg/mL), using the standard curve (Equation (2)):

y = 0.0009x + 0.613, R^2^ = 0.994 (2)

y = absorbance at 510 nm and x = concentration of total phenolic content rutin equivalent mg/mL.

### 2.6. In-Vitro Antioxidant Activity

The 2,2-diphenyl-1-picrylhydrazyl (DPPH) scavenging activity was used as described by Al-Bayati et al. (2023) [34]. For the reaction reagent, DPPH was dissolved in methanol at a concentration of (0.04 g/mL). The reaction was performed by dissolving plant extract in methanol at a concentration of (0.01 g/mL). An aliquot of 1 mL of the plant extract solution was mixed with 3 mL of DPPH and completed to a final volume of 10 mL using methanol, then allowed to stand in darkness for 30 min. Absorbance was measured at a wavelength of 517 nm. Ascorbic acid was used as a reference for comparison (Sigma Aldrich, Darmstadt, Germany). A calibration curve of ascorbic acid was used for the calculation of the effective concentration required for scavenging DPPH free radicals (% inhibition Equation (3)).

% inhibition = [(A control − A sample)/A control] × 100(3)

where: A control = absorbance of the control sample, and A (sample) = absorbance of the sample.

### 2.7. UHPLC-MS/MS Methodology

#### Instrumentation and MS Parameters

The UHPLC coupled with Impact II QTOFMS Bruker Daltonik (Bremen, Germany) was used for screening the compounds of interest using the same method previously described by Al-Bayati et al. (2023) [34]. The instrument operation conditions were as follows: Apollo II ion funnel electrospray source, capillary voltage (2500 V), nebulizer gas (2 bar), and nitrogen dry gas at a flow rate of 8 L/min (200 °C). The mass accuracy was <1 ppm; with Full Sensitivity Resolution (50000 FSR) and the TOF repetition rate of 20 kHz.

Bruker Solo 2.0_C-18 UHPLC column (100 mm × 2.1 mm × 2.0 μm) was used for chromatographic separation at a flow rate of 0.51 mL/min (40 °C). The mobile phase consists of (A: 0.05% formic acid in water), and (B: acetonitrile). Gradient elution was used as follows: 0–27 min linear gradient from 5–80% B; 27–29 min 95% B; 29.1 min 5% B. The total analysis time was 35 min in positive mode and 35 min in negative mode, with an injection volume of 3 µL.

MC and DES samples stock solutions were prepared by dissolving an appropriate amount of the plant extract in dimethyl sulfoxide-DMSO (analytical grade), then diluted with acetonitrile to complete 50 mL, then centrifugation was performed at 4000 rpm was applied for 2 min. All the other reagents, Acetonitrile, methanol, water, and formic acid used were LC-MS grade.

Sample preparation: 100 µL of each sample has been dissolved in 900 µL of MeOH. A 1.0 mL was transferred to an autosampler and injected. Identification of phenols and flavonoid compounds was based on the retention time (Rt), mass spectrum (*m*/*z*), and molecular formula, compared to a previously developed integrated library of natural compounds.

### 2.8. Nicotine Content Determination

The nicotine content was determined according to the process described by Kheawfu et al. in their 2021 study [35]. Briefly, each obtained extract was analyzed by UHPLC coupled with Impact II QTOFMS Bruker Daltonik (Bremen, Germany). Bruker Solo 2.0_C-18 UHPLC column (100 mm × 2.1 mm × 2.0 μm). A linear elution mobile phase composed of Sodium acetate, methanol, and trimethylamine (88:12:0.5 *v*/*v*) (pH = 4.2) was used. The mobile phase elution was adjusted to a flow rate of 1 mL/min and measured at UV = 259 nm. The total analysis time was 20 min in positive mode.

Nicotine standard (AccuStandard^®^, Inc., New Haven, CT, USA) was used for establishing the calibration curve at concentrations ranging from (0.10–2.00 µg/mL) in water and was used for the calculation of nicotine content in plant extracts as ppm values.

Sample preparation for UHPLC-MS/MS analysis: (A) 500 µL from MC or DES extract samples were diluted with 500 µL methanol, then the solution was centrifuged at 4000 rpm for 2.0 min. Next, 1.0 mL was transferred to the autosampler and 3.0 µL was injected into the system (D.f. = 2). (B) Then, 50 µL was taken from sample (A), and diluted with 1950 µL of methanol. Next, 1.0 mL was transferred to the autosampler and 3.0 µL was injected into the system (D.f. = 40). (C) Then, 100 µL was taken from sample (B), and diluted with 1900 µL of methanol. Next, 1.0 mL was transferred to the autosampler and 3.0 µL was injected into the system (D.f. = 20), (D.f.total = 2 × 40 × 20).

## 3. Results

In the present study, the efficiency of the synthesized DES to recover phenolic compounds from *N. glauca* leaf was tested using two representative phytochemical indices such as TPC and TFC. The structure of DES significantly influences their physicochemical properties, impacting their extraction efficiency. In this study, DES were created using CC as a hydrogen bond acceptor (HBA) and MA as a hydrogen bond donor (HBD), with water added at 10%, 30%, and 70% concentrations. All the solvents remained stable without precipitating during preparation, extraction, and analysis. Viscosity and polarity are crucial for DES’s efficiency and can be modified by adding water. Adding water helps lower viscosity and enhances tunability. However, excessive water can weaken interactions within the DES and with extracted components, reducing efficiency. The optimal water addition (25–30%) improves extraction, while higher amounts (40–75%) can diminish it. Previous studies, such as Dai et al.’s 2013 study [36], noted that significant water addition can alter polarity and disrupt hydrogen bonds in DES. Additionally, the biological activities of extracts using DES were compared to those obtained with traditional solvents like methanol.

### 3.1. Total Phenol Content

The extract that obtained from the MC method was shown to contain less total phenolic compounds (0.1194 ± 0.009 mg/mL equivalent to gallic acid), compared to the DES extracts which showed similar total phenol content for the three prepared extract ratios with almost no significant difference between the three tested DES ratios (30, 70, and 90%) corresponding to an average of 0.312 ± 0.13 mg/mL (equivalent to gallic acid) (Table 1). This finding is in close accordance with the results reported by others who found that carboxylic-based DES have a high ability to extract phenolics from plants [37,38,39]. The high extraction efficiency of DES may be due to the hydrogen bond interaction between the phenolic compounds and the DES’s components.

### 3.2. Total Flavonoid Content

Total flavonoid content was also analyzed to compare the extraction efficiency of DES and MC, as well as to evaluate the difference between DES with varying water content (Table 2). The DES with the lowest water content (10%) resulted in the highest total flavonoid content, which was 7–8 times higher than the other two DES and about 84 times higher than MC. The extraction pattern of flavonoids by the tested DES with different water content and with MC was found to be similar to that of the phenolic extraction. It can be concluded that extraction yields not only depend on solvent types but also on the physical and chemical characteristics of the samples. It should be mentioned that flavonoids themselves can also act as hydrogen bond donors, potentially competing with the HBD used in DES preparation. However, in this study, only one class of DES were tested, so this factor had no effect.

### 3.3. Antioxidant Activity (DPPH Assay) for N. glauca Leaf Extracts

Maceration is a traditional and one of the most ancient extraction processes applied for the extraction of bioactive substances such as phenolic compounds. Although maceration is a time-consuming method, it has been reported to be adequate and subsequent for the recovery of antioxidants from various plant materials. The Antioxidant capacities of the extracts obtained by DES and MC were measured using DPPH. The most concentrated DES extract (90%) exhibited the highest % Inhibition of free radical activity at 33%, equivalent to ascorbic acid. In contrast, the other DES ratios and MC exhibited weak antioxidant activities and failed to change the color of the DPPH reagent from dark purple to pale yellow, rendering the antioxidant test ineffective for these samples. It can be concluded that the nature of the extraction solvent greatly influences the type of extractives obtained and their antioxidant activity. Many reports in the literature demonstrated that extracts prepared in organic acid-based DES show better antioxidant activity than those prepared in aqueous, methanolic, and ethanolic extracts. Pavic et al., (2019) [40] found that Ruta leaf extracts prepared in NADES of CC and citric acid in a 2:1 molar ratio had the highest phenolic content and the highest DPPH radical scavenging activity. Additionally, Bakirtzi et al., (2016) [41] reported the highest reducing power in sage extracts obtained using NADES of lactic acid and CC in a 3:1 molar ratio.

### 3.4. Identification of Phenols Using UHPLC-MS/MS Analysis

#### 3.4.1. Methanol Conventional Extraction (MC)

A total of twenty-three different phenolic components have been detected in the MC extract using the LC-MS/MS analysis, and the integrated natural compounds library. The retention time (Rt)mass-to-charge ratio (*m*/*z*), and molecular formula for the detected compounds (positive and negative ion modes) are listed in Table 3. A total of fourteen compounds were detected- in the negative mode, and nine were detected in the positive mode.

**Figure 1:**
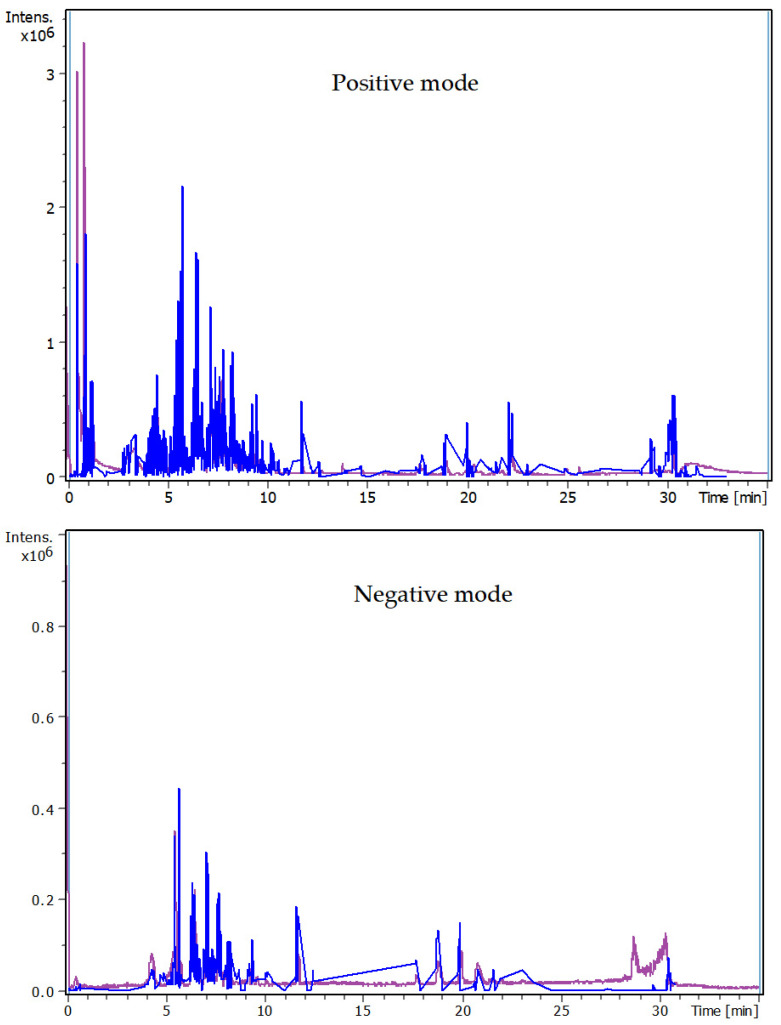
Total ion chromatograms for all compounds detected in *N. gluca* MC extract.

**Figure 2:**
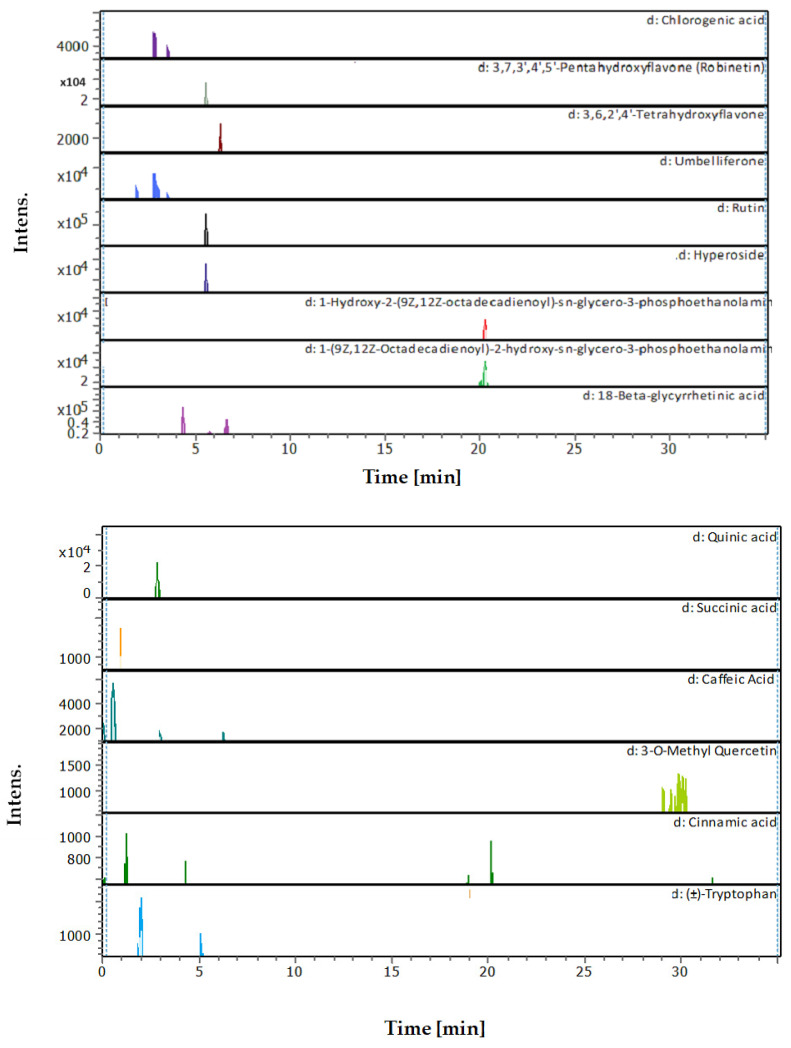

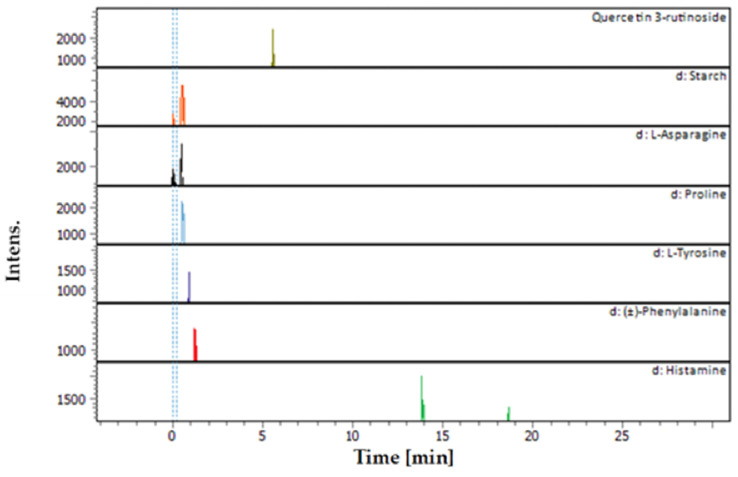
The UHPLC-chromatograms show peaks and retention time of each compound detected in *N. glauca* prepared by MC extraction.

Figure 1 shows the total ion chromatogram for all compounds detected in the MC leaves extracts. The UHPLC-chromatograms which display the peaks and retention time of each compound detected in the extract are shown in Figure 2. The mass spectrum (*m*/*z*) and fragments for each compound detected in the MC extract are presented in Appendix A.

#### 3.4.2. Deep Eutectic Extraction (DES)

A total of twenty-three different phenolic components have been detected in the DES extract using the UHPLC-MS/MS analysis, and the integrated natural compounds library. The Rt, *m*/*z*, and molecular formula for the detected compounds (positive and negative ion modes) are listed in Table 4. A total of ten compounds were detected in the negative mode, and thirteen were detected in the positive mode.

**Figure 3:**
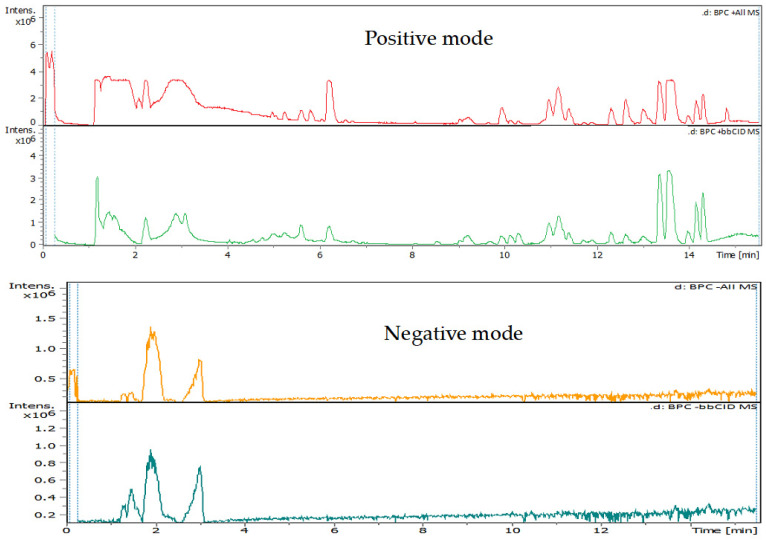
Total ion chromatograms for all compounds detected in *N. glauca* DES extract.

**Figure 4:**
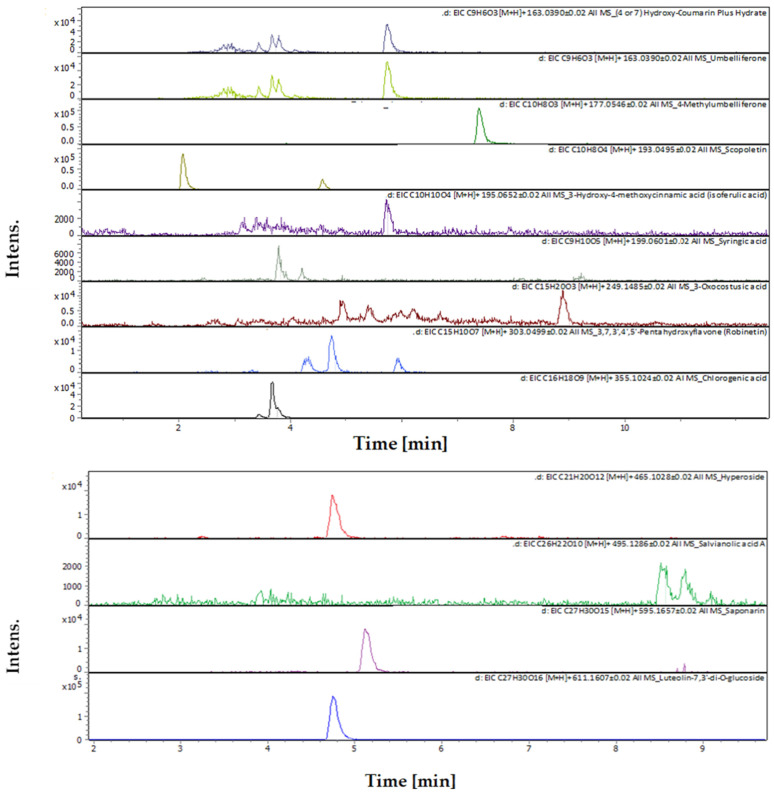

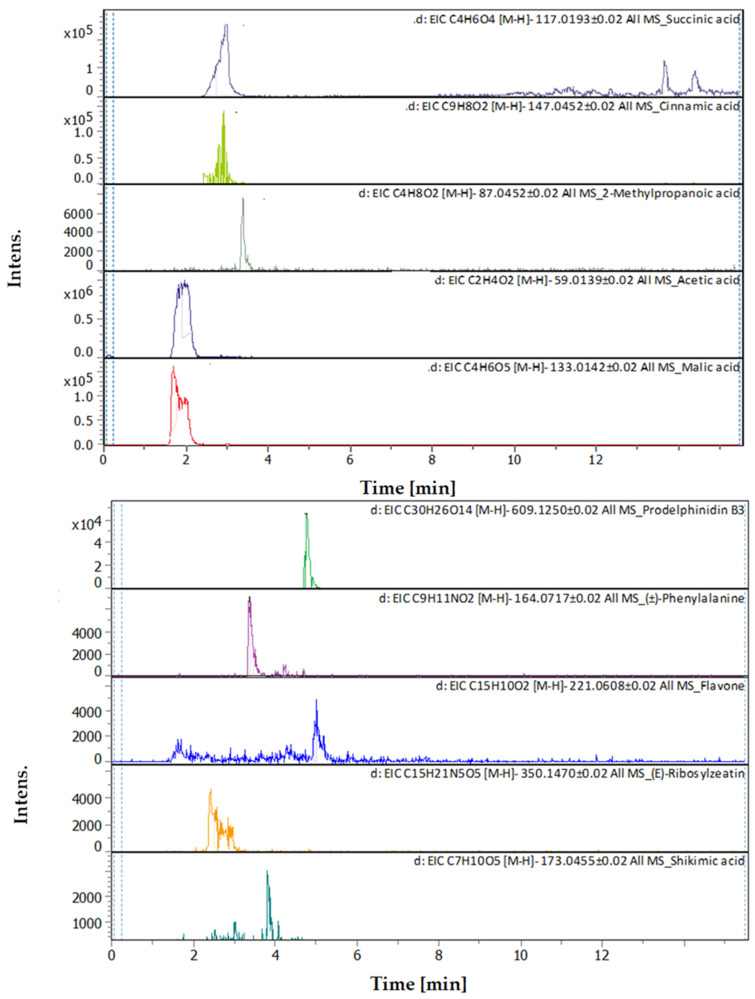
The UHPLC-chromatograms show peaks and retention time of each compound detected in *N. glauca* DES extract.

Figure 3 shows the total ion chromatogram for all compounds detected in the DES leaf extracts. The UHPLC-chromatograms showing peaks and retention time of each compound detected in the extract are shown in Figure 4. The Mass spectrum (*m*/*z*) and fragments for each compound detected in the MC extract are available in Appendix B.

#### 3.4.3. Identification and Quantification of Nicotine

**Figure 5:**
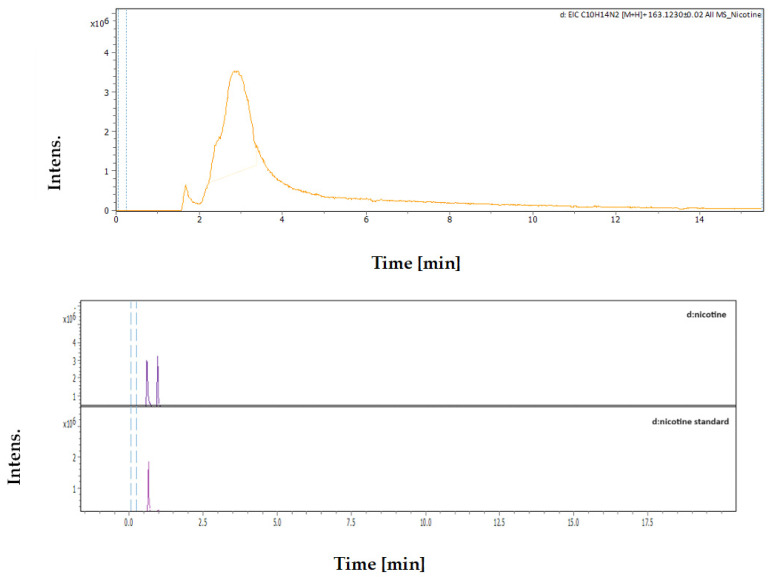
The UHPLC-chromatogram and mass spectra show peaks and retention time of nicotine detected in *N. glauca* extract.

The identification of nicotine in the extract samples was performed using a multiple external standards method using the UHPLC-MS/MS system, based on the retention time, *m*/*z*, and molecular formula as shown in Table 5 and Figure 5.

The results indicated that the concentration of nicotine in the MC extract was 635.07 ppm (µg/mL) (0.064%), while the concentration in the DES extract was 1194.91 ppm (µg/mL) (0.119%) (i.e., almost twice). This suggests that the DES extraction method yielded a higher concentration of nicotine compared with the MC method, proving that the DES method is more effective for the extraction of this alkaloid. The levels of nicotine extracted using the two techniques are illustrated in Table 6.

Alkaloids are the dominant class of constitutive secondary chemicals in *N. glauca*, but in contrast to most Nicotiana species, the major alkaloid in *N. glauca* tissues is anabasine [42]. To the best of our knowledge, limited work was performed to study the content of nicotine in the species *N. glauca*. Comparing our findings with the other extraction methods mentioned below for the species *N. tabacum*, both extracts (MC and DES) showed a lower content of nicotine than the previously published data. A similar investigation was performed by Tayoub et al. (2015) [43] aimed at measuring nicotine levels in the leaves of seven different varieties of *N. tabacum*, which were cultivated in Syria. The study reported that nicotine is naturally present in a concentration of 0.3 to 3%. Tantullavetch et al. (2007) [44] reported that nicotine extraction yield from tobacco leaves with acid-base extraction was approximately 4.2%. Kheawfu et al., (2021) [35] studied the effect of the use of different extraction solvents on the yield of nicotine extracted from *N. tabacum* leaves collected from different positions on the stem. Findings using the acid-base extraction method presented the highest nicotine content (43.28–63.17%) compared to the maceration extraction method using water (1.27–12.07%) and ethanol (10.78–16.99%).

In a study by Banožić et al., (2021) [45] nicotine was extracted using a microwave-assisted extraction method. The extraction took place under different conditions and temperatures. The study’s findings showed that nicotine is the dominant compound with concentrations in the range 1.512–5.480, 1.886–3.709, 2.628–4.840, and 0.867–1.783% for leaves, scrap, dust, and midrib extracts, respectively. Therefore, the concentration of nicotine is highly dependent on tobacco species type, variety, growing, and environmental conditions, in addition to variations in the extraction techniques used.

## 4. Discussion

Several studies reported high toxicity of the plant *N. glauca* caused by its alkaloid content, namely nicotine and other derivatives [46,47]. The use of *N. glauca* as an anti-jaundice plant among herbalists and traditional healers was reported in Jordanian folk medicine [48]. Nevertheless, few reports investigated the phytochemical composition of the wild species grown in Jordan. Researchers detected the presence of different phenolic compounds in tobacco plants such as kaempferol-3-O-rutinoside, quercetin-3-O-rutinoside, in addition to the main components of tobacco polyphenols are chlorogenic acid and rutin [4,49,50]. In this study, the UHPLC-MS/MS analysis revealed the presence of variations in the detected compounds between the two extraction methods used. Mainly, 3,7,3′,4′,5′-Pentahydroxyflavone (Robinetin), Chlorogenic acid, Hyperoside, Rutin, and Umbelliferone, were detected in both extracts. Whereas (4 or 7) Hydroxy-Coumarin Plus Hydrate, 3-Hydroxy-4-methoxycinnamic acid (isoferulic acid), 3-Oxocostusic acid, 4-Methylumbelliferone, Luteolin-7,3′-di-O-glucoside, Salvianolic acid A, Saponarin, Scopoletin, and Syringic acid, were detected in the DES extract only. On the other side, 1-(9Z,12Z-Octadecadienoyl)-2-hydroxy-sn-glycero-3-phosphoethanolamine,18-Beta glycyrrhetinic acid, 1-Hydroxy-2-(9Z,12Z-octadecadienoyl)-sn-glycero-3-phosphoethanolamine, and 3,6,2′,4′-Tetra hydroxy flavone, were detected in the MC extract only.

Our work on the total content of phenolic phytocomponents showed the MC extract contained fewer total phenols and flavonoid content compared to the DES extract. These findings were expected, as previous studies highlighted the advantages of using NaDES as a green solvent in the extraction of phytochemicals, including enhanced extraction yield, and additional environmental benefits [28,29]. The results of the antioxidant DPPH test revealed that both extraction methods have weak antioxidant activities, except for the concentrated (90%) DES extract which showed a moderated antioxidant activity compared to ascorbic acid. The (90%) DES extract was also found to contain the highest total flavonoid content among the other extract samples. These findings are in correlation with phenols and flavonoid content, which are the most contributing natural compounds for antioxidant activity. Our findings agree with a previous study by Trifa, et al. (2020) [51], investigating *N. glauca* extract collected from central Algeria, which showed good antioxidant activity in the ethyl acetate and n-butanol fractions, which was related to the content of polyphenols. Similarly, Sumengen et al. (2023) [52] conducted a study to investigate the phytochemical composition of *N. glauca* methanol leaf extract collected from Northern Cyprus. Findings showed that *N. glauca* methanolic extract had the highest antioxidant activity determined using the DPPH test and were correlated to their content of phenols and flavonoids. The latter compared his findings with others’ previous work, showing that variations in the antioxidant effects are expected, due to variations in the phytochemical composition. These variations may occur due to several factors, such as growing conditions, environmental variations, stage of plant development, season of collection, and geographical origin, in addition to methods of extraction and solvent used.

It was previously found that the acid-base extraction method contains the highest nicotine content, as this method aids in solubilizing the alkaloids by converting them to the salt form, which enhances their solubility in polar solvents [35]. It was concluded that methods of extraction and solvent used, plant part, and many other variables would largely be affecting nicotine content, in addition to other phytocomponents obtained in the plant extract.

The findings of the presented study showed that the MC extract contains 635.07 ppm of nicotine compared to 1194.91 ppm of nicotine found according to the DES extraction method. In agreement with other published work, Kheawfu et al. (2021) [35] found that extraction with water for 24 h gave the highest amount of nicotine. Whereas Puripattanavong et al. (2013) [53] suggested that using methanol and ethanol gave the highest yield percentage of nicotine extraction from *N. tabacum* leaves compared with other solvents and extraction media. A similar work by Massadeh et al. (2022) [4] screening the phytochemical constituent in the leaves of *N. glauca* collected from the north region in Jordan [4]. Using the UPLC-MS and GC-MS analysis, anabasine was detected as the major alkaloid, while nicotine was not detected in their studied extract.

## 5. Conclusions

In conclusion, *N. glauca* contains a substantial content of valuable phenols and flavonoids, with a low amount of nicotine compared to other *Nicotiana* species. Moreover, results demonstrated that the extract that resulted from the MC method has the lowest content of the detected phytochemicals compared to the extracts that resulted from the DES method. Similarly, the antioxidant activity of the prepared extracts showed no effects of all extracts except for the concentrated extract from the DES extraction (90%), which showed the highest activity (33%) relative to ascorbic acid.

These results emphasize the critical importance of adopting green chemistry techniques, not only for their environmental benefits but also for their superior efficiency in producing higher yields of valuable phytoconstituents. In addition, optimization of the extraction procedure, as well as plant selection and preparation was shown to largely influence the content of secondary phytocompounds. Despite the work performed and published on the content of alkaloids in the study plant species, further investigation of the proportions of other alkaloids found in *N. glauca* is still required.

## Figures and Tables

**Table 1:** Total phenols content in MC and DES extracts at three extract ratios.

Extraction Media	mg/mL ± SD (Equivalent to Gallic Acid)
DES 30%	0.326 ± 0.11
DES 70%	0.300 ± 0.03
DES 90%	0.311 ± 0.02
MC	0.119 ± 0.01

**Table 2:** Total flavonoid content in MC and DES extracts at three extract ratios.

Extraction Media	mg/mL ± SD (Equivalent to Rutin)
DES 30%	0.128 ± 0.03
DES 70%	0.115 ± 0.14
DES 90%	0.928 ± 0.09
MC	0.011 ± 0.01

**Table 3:** UHPLC-MS/MS analysis (positive and negative modes) showing all components detected in *N. glauca* MC extract based on retention time (Rt), Mass-to-charge ratio (*m*/*z*), and molecular formula.

#	Rt [min]	*m*/*z* Meas.	M Meas.	Ions	Name	Molecular Formula
1	0.61	131.04612	132.05339	[M–H]^−^	L-Asparagine	C_4_H_8_N_2_O_3_
2	0.62	114.05616	115.06344	[M–H]^−^	Proline	C_5_H_9_NO_2_
3	0.97	180.06594	181.07322	[M–H]^−^	L-Tyrosine	C_9_H_11_NO_3_
4	1	117.01933	118.02661	[M–H]^−^	Succinic acid	C_4_H_6_O_4_
5	1.28	147.04508	148.05236	[M–H]^−^	Cinnamic acid	C_9_H_8_O_2_
6	1.28	164.0718	165.07908	[M–H]^−^	(±)-Phenylalanine	C_9_H_11_NO_2_
7	2.03	203.08226	204.08954	[M–H]^−^	(±)-Tryptophan	C1_1_H_12_N_2_O_2_
8	2.9	191.05607	192.06335	[M–H]^−^	Quinic acid	C_7_H_12_O_6_
9	2.96	355.10248	354.09521	[M+H]^+^	Chlorogenic acid	C_16_H_18_O_9_
10	3.05	179.03487	180.04214	[M–H]^−^	Caffeic Acid	C_9_H_8_O_4_
11	5.07	163.03979	162.03251	[M+H]^+^	Umbelliferone	C_9_H_6_O_3_
12	5.12	203.08228	204.08955	[M–H]^−^	(±)-Tryptophan	C_11_H_12_N_2_O_2_
13	5.57	609.1455	610.15278	[M–H]^−^	Quercetin 3-rutinoside	C_27_H_30_O_16_
14	5.61	303.05014	302.04287	[M+H]^+^	Robinetin	C_15_H_10_O_7_
15	5.61	465.10293	464.09566	[M+H]^+^	Hyperoside	C_21_H_20_O_12_
16	5.62	611.16099	610.15353	[M+H]^+^, [M+Na]^+^	Rutin	C_27_H_30_O_16_
17	6.31	179.05592	180.0632	[M–H]^−^	Starch	C_6_H_12_O_6_
18	6.37	287.0557	286.04842	[M+H]^+^	3,6,2′,4′-Tetrahydroxyflavone	C_15_H_10_O_6_
19	9.1	315.05061	316.05788	[M–H]^−^	3-O-Methyl Quercetin	C_16_H_12_O_7_
20	21.09	478.28898	477.2817	[M+H]^+^	1-Hydroxy-2-(9Z,12Z-octadecadienoyl)-sn-glycero-3-phosphoethanolamine (NMR)	C_23_H_44_NO_7_P
21	22.31	478.28916	477.28189	[M+H]^+^	1-(9Z,12Z-Octadecadienoyl)-2-hydroxy-sn-glycero-3-phosphoethanolamine (NMR)	C_23_H_44_NO_7_P
22	22.49	471.35137	470.34409	[M+H]^+^	18-Beta-glycyrrhetinic acid	C_30_H_46_O_4_
23	28.62	221.15517	222.16244	[M–H]^−^	Histamine	C_10_H_18_N_6_

**Table 4:** UHPLC-MS/MS analysis (positive and negative modes) showing all components detected in *N. glauca* DES extract based on retention time (Rt), Mass (*m*/*z*), and molecular formula.

#	Rt [min]	*m*/*z* Meas.	M Meas.	Ions	Name	Molecular Formula
1	1.65	133.00998	134.0174	[M–H]^−^, [M–H H2O]^−^	Malic acid	C_4_H_6_O_5_
2	1.91	59.01128	60.01856	[M–H]^−^	Acetic acid	C_2_H_4_O_2_
3	2.39	350.14397	351.15125	[M–H]^−^	(E)-Ribosylzeatin	C_15_H_21_N_5_O_5_
4	2.96	117.01473	118.022	[M–H]^−^	Succinic acid	C_4_H_6_O_4_
5	3.32	147.04052	148.0478	[M–H]^−^	Cinnamic acid	C_9_H_8_O_2_
6	3.35	87.04212	88.04939	[M–H]^−^	2-Methylpropanoic acid	C_4_H_8_O_2_
7	3.36	164.06681	165.07409	[M–H]^−^	(±)-Phenylalanine	C_9_H_11_NO_2_
8	3.69	163.038920	162.031640	[M+H]^+^	Umbelliferone	C_9_H_6_O_3_
9	3.7	355.102500	354.095190	[M+H]^+^, [M+K]^+^, [M+Na]^+^	Chlorogenic acid	C_16_H_18_O_9_
10	3.79	173.04114	174.04841	[M–H]^−^	Shikimic acid	C_7_H_10_O_5_
11	3.8	199.057710	198.050440	[M+H]^+^	Syringic acid	C_9_H_10_O_5_
12	4.6	193.049450	192.042170	[M+H]^+^	Scopoletin	C_10_H_8_O_4_
13	4.76	465.102820	464.095540	[M+H]^+^	Hyperoside	C_21_H_20_O_12_
14	4.77	303.050010	302.042740	[M+H]^+^	Robietin	C_15_H_10_O_7_
15	4.77	611.160240	610.152970	[M+H]^+^	Luteolin-7,3′-di-O-glucoside	C_27_H_30_O_16_
16	4.91	609.1281	610.13537	[M–H]^−^	Prodelphinidin B3	C_30_H_26_O_14_
17	5	221.05958	222.06686	[M–H]^−^	Flavone	C_15_H_10_O_2_
18	5.15	595.165470	594.158150	[M+H]^+^, [M+Na]^+^	Saponarin	C_27_H_30_O_15_
19	5.74	195.065060	194.057780	[M+H]^+^	3-Hydroxy-4-methoxycinnamic acid (isoferulic acid)	C_10_H_10_O_4_
20	7.39	163.039120	162.031850	[M+H]^+^	(4 or 7) Hydroxy-Coumarin Plus Hydrate	C_9_H_6_O_3_
21	7.41	177.054460	176.047190	[M+H]^+^	4-Methylumbelliferone	C_10_H_8_O_3_
22	8.54	495.125630	494.118360	[M+H]^+^	Salvianolic acid A	C_26_H_22_O_10_
23	11.6	249.148000	248.140730	[M+H]^+^	3-Oxocostusic acid	C_15_H_20_O_3_

**Table 5:** UHPLC-MS/MS analysis for nicotine detected in *N. glauco* extracts based on retention time.

Rt [min]	*m*/*z* Meas.	M Meas.	Ions	Name	Molecular Formula
2.89	163.12293	324.2313	[M+H+H]^2+^	Nicotine	C_10_H_14_N_2_

**Table 6:** Area under the curve and concentration of nicotine (ppm) in the MC and DES extracts based on multiple external standards method.

Sample	MC Extract	DES Extract
Area of Nicotine in Sample	2,526,713	4,192,477
Concentration of Nicotine	635.07 ppm	1194.91 ppm

## Data Availability

Data are contained within the article.
